# Topical regimens for the management of uremic pruritus

**DOI:** 10.3389/fmed.2026.1857124

**Published:** 2026-08-12

**Authors:** Tingting Zhu, Fumin Fang, Dongyun Lei, Guoqiang Zhang, Mao-Qiang Man

**Affiliations:** 1 Department of Dermatology, The First Affiliated Hospital of Soochow University, Suzhou, China; 2 Department of Dermatology, Tianjin Academy of Traditional Chinese Medicine Affiliated Hospital, Tianjin, China; 3 Department of Dermatology, The First Hospital of Hebei Medical University, Shijiazhuang, China; 4 Hebei Technical Innovation Center for Dermatology and Medical Cosmetology Technology, Shijiazhuang, Hebei, China

**Keywords:** emollient, hemodialysis, pruritus, stratum corneum hydration, uremic pruritus

## Abstract

Uremic pruritus (UP), also known as chronic kidney disease-associated pruritus, is a distressing condition affecting patients with end-stage renal disease. Characterized by persistent itching, it significantly impacts patients’ quality of life, leading to sleep disturbances, emotional distress, and decreased overall well-being. Although the exact pathophysiology remains uncertain, immune dysregulation, reduced stratum corneum hydration, and altered neurogenic inflammation contribute to UP. Consequently, treating UP poses a significant challenge. Among therapeutic regimens, topical regimens have been shown to effectively alleviate UP. Various topical formulations, including urea-based, physiological lipids, natural oils, capsaicin, anesthetics and glycerin-containing emollients, have demonstrated benefits in amelioration of pruritic symptoms. Clinical studies suggest that consistent application of emollients can minimize skin dryness and relieve pruritus. This review summarizes key clinical findings on the topical therapeutic regimens for UP.

## 1 Introduction

Pruritus is a common symptom caused by both cutaneous and extracutaneous conditions (1, 2). Uremic pruritus (UP) occurs in patients with end-stage renal dysfunction. Although its pathogenesis remains unclear, several hypotheses have been proposed, including uremic toxin accumulation, immune dysregulation, peripheral neuropathy, dry skin, hyperparathyroidism, and opioid imbalance (3, 4). For example, individuals with renal dysfunction have higher circulating levels of IL-6, histamine and cutaneous calcium phosphate (5–9). The latter also increases IL-6 levels in the skin, while IL-6 can phosphorylate extracellular signal-regulated kinase in the dorsal root ganglion, provoking pruritus (9). Moreover, histamine is a well-known pruritogenic mediator (10). Furthermore, individuals with renal dysfunction exhibit high levels of serotonin (8), which binds to its receptor, HTR7, resulting in activation of transient receptor potential ankyrin 1, leading to the development of serotonergic pruritus (11). Additionally, the prevalence of xerosis ranges from 50% to 85% in subjects with end stage renal disease (12). Reduced stratum corneum hydration levels increase cutaneous mast cell infiltration and expression levels of histamine and proinflammatory cytokines in addition to increase nerve growth factor and acetylcholine in the skin (13, 14). In addition to reduced stratum corneum hydration levels, skin surface pH is elevated in subjects with end-stage renal disease. The elevated stratum corneum pH increases serine protease and proteinase-activating receptor 2 activities, consequently resulting in pruritus (14). However, study shows that the circulating levels of urea, creatinine, calcium, phosphorus, alkaline phosphatase, and parathyroid hormone are unlikely associated with uremic pruritus (15). Nonetheless, multiple factors contribute to the pathogenesis of uremic pruritus. The uncertainty surrounding its underlying mechanisms presents a significant challenge in managing uremic pruritus (UP). However, evidence suggests that several therapeutic approaches can help alleviate UP, including optimization of hemodialysis techniques, phototherapy, systemic treatments, and topical therapies. This review provides a brief overview of the topical therapeutic regimens of UP based on a literature search on and Google scholar from inception to November 2025. The search terms included “pruritus,” “itch,” “kidney,” “hemodialysis,” “emollient,” “cream,” and “lotion” on PubMed and Google Scholar. Additionally, only studies published in English were reviewed. Studies on pruritus not related to kidney dysfunction or published in languages other than English were excluded.

Evidence shows that various topical agents are available for ameliorating uremic pruritus (UP) through mechanisms such as improving stratum corneum hydration, reducing inflammation, activating transient receptor potential vanilloid 1 (TRPV1), and providing local anesthesia (Table 1) (16–53). However, some studies did not show any correlation between pruritus and either stratum corneum hydration or epidermal permeability barrier in patients with UP (54–56).

**TABLE 1:** Topical therapeutic regimens.

Emollients	Type of study	Treatment	Outcome	Safety	Ref.
Mineral oil and vitamin E	Non-randomized, pretest-post-test quasi-experimental study	Control and intervention groups consisted of 60 patients each. A mixture of mineral oil and vitamin E was applied to the itchy area and gently massaged for 15 min once daily for 10 consecutive days. No intervention with topical emollient served as the controls	Intervention significantly lowered VRS (23.7% vs. 1.3%)	NR	(16)
Mineral oil	Randomized, pretest-post-test quasi-experimental study	30 cases were treated with chilled and 31 cases treated un-chilled mineral oil at least once daily for 3 weeks. Controls were 32 untreated patients.	Both chilled and un-chilled mineral significantly decreased ISS	3 out of 96 had skin rash, not sure whether that were product-related.	(17)
Mineral oil	Randomized pretest-post-test quasi-experimental study	39 controls and 38 cases in the intervention groups were enrolled. The intervention group was treated with mineral oil three times daily for 4 weeks.	Significant reduction (43%) in VAS (*p* < 0.001 vs. controls)	NR	(18)
Mineral oil	Non-randomized, pretest-post-test quasi-experimental study	Control and intervention groups consisted of 30 patients each. Chilled mineral oil was applied to the itching areas once for 15 min prior to each hemodialysis, over a 30-days period.	VAS 5.71 vs. 3.17; ISS 15.22 vs. 8.85, *p* < 0.01 for both.	NR	(19)
Glycerol and paraffin	Randomized, double-blind, placebo-controlled study	Phase I. 15% glycerol and 10% paraffin (G/P) cream in an emulsion was applied to one lower leg of 99 subjects twice daily for 7 days, while emulsion alone was applied to the other leg. Phase II. Both legs were treated with G/P cream twice daily for 56 days.	In Phase I, G/P significantly improved dryness (*p* < 0.0001 vs. control); In phase II, 75% reduction in VAS.	Exacerbations of pruritus in 2 cases; local burning or hyperesthesia in 2 cases, and erythema in 1 case.	(20)
Glycerol and paraffin	Randomized, double-blind, placebo-controlled study	A total of 187 subjects completed the study. G/P cream was applied ad libitum by the patients based on their lesion status, tolerance, and acceptability of the product for 84 days.	Decreased VAS from 10.4 ± 1.9 to 7.5 ± 1.4	Irritation or erythema in 2 cases; exacerbated pruritus in 3 cases; vesicles at the application site in 1 case; allergic dermatitis and dyshidrosis in 1 case each.	(21)
Glycerol and paraffin	Randomized controlled trial with a two-group pretest–post-test quasi-experimental study	30 patients were treated with G/P cream twice daily for 28 days	Decreased VAS from 5.26 to 3.6 on day 7, and 0.33 on day 28	NR	(22)
Mustard oil	Randomized controlled trial with a two-group pretest–post-test quasi-experimental study	30 patients were treated with mustard oil twice daily for 28 days	Decreased VAS from 5.76 to 4.03 on day 7, and 0.50 on day 28	NR	(22)
Sweet almond oil	Randomized, controlled trial with a two-group pretest-post-test quasi-experimental study	22 patients received 5–10 mL of the oil, which was applied topically to either the affected itching areas or the whole body once daily for 2 weeks. 20 patients received no oil treatment.	Pruritus-specific QoL decreased by 18.6% in the oil-treated group and increased by 4.3% in the controls (*p* < 0.001)	NR	(23)
Violet oil	Randomized, controlled trial with a two-group pretest-post-test quasi-experimental study	25 patients were massaged with 5 ml of violet oil for 7 min, once every other day, for a total of six sessions (2 weeks), and 27 cases received massage without oil.	In the controls, dry score decreased from 1.55 ± 0.97 to 1.17 ± 0.84, and VAS from 2.77 ± 0.69 to 2.15 ± 3.80; In the oil group, dry score decreased from 1.94 ± 0.99 to 0.82 ± 0.40, and VAS from 1.68 ± 0.47 to 0.94 ± 0.46	No adverse events	(24)
Virgin coconut oil	Non-randomized trial with a two-group pretest-post-test quasi-experimental study	36 patients were treated topically with virgin coconut oil twice daily for 2 weeks.	36% of patients itching grade 2 and 53% grade 3 at baseline; 48% itching grade 2 and no grade 3 after the treatment.	NR	(25)
Olive oil	Non-randomized trial with a two-group pretest-post-test quasi-experimental study	36 patients were treated topically with olive oil twice daily for 2 weeks.	83% of patients itching grade 2 and 14% grade 3 at baseline; 25% itching grade 2 and no grade 3 after the treatment.	NR	(25)
Dill oil	Randomized, double-blind three-arm controlled trial	37 patients each were treated with either dill oil or sesame oil twice daily for 1 month. 37 untreated patients served as controls	Dill oil decreased VAS (−3.43 ± 3.22 vs. −17.24 ± 8.93, *p* < 0.001 vs. controls), and improve quality of life (14.18 ± 7.89 vs. 1.92 ± 2.86, *p* < 0.001 vs. controls)	No adverse events	(26)
Ostrich oil	Non-randomized, placebo controlled trial	35 patients were massaged with 1 ml of ostrich oil for 10 min once daily for 1 month. 34 cases massaged without vaseline oil served as controls	Ostrich oil improved ISS (5.13 ± 1.42 vs. 6.14 ± 1.39, *p* < 0.05 vs. controls)	NR	(27)
Chia seed oil	Non-randomized trial with a two-group pretest-post-test quasi-experimental study	Total 5 patients with dry skin (4 had renal dysfunction and 1 had diabetes) were included. One side of body was treated with emollient containing 4% chia seed oils for 8 weeks and the contralateral side was treated with a vehicle	Compared to vehicle, chia seed oil significantly increased skin hydration, without significant improvement in pruritus.	No adverse events	(28)
Peppermint oil	Non-randomized trial with a two-group pretest-post-test quasi-experimental study	8 patients were treated with 5% peppermint oil for 2 weeks.	Decreased 5-D IS from 14.94 ± 5.66 to 6.96 ± 4.52, *p* < 0.001	Burning sensation in 4 cases.	(29)
Petrolatum	Non-randomized trial with a two-group pretest-post-test quasi-experimental study	8 patients were treated with petrolatum for 2 weeks.	No significant changes in 5-D IS. 10% reduction in pruritus severity (*p* = 0.01)	NR	(29)
Calcipotriol	Randomized, double-blind, placebo-controlled study	31 patients were treated with 0.005% calcipotriol twice daily for 4 weeks and 31 placebo-treated served as controls	VAS decreased from 6.61 ± 2.49 to 1.45 ± 1.58 in calcipotriol group vs. 6.74 ± 2.35 to 4.26 ± 2.32 in controls	No adverse events	(30)
Capsaicin	Randomized, double-blind, placebo-controlled study	17 patients were treated with 0.025% capsaicin cream (10 cases) or placebo (7 cases) was applied to pruritic areas 4 times daily for 4 weeks. Then the two groups switched treatments and continued for another 4 weeks.	Capsaicin induced a significant reduction in pruritus (*p* < 0.001)	No significant differences in adverse events, including mild burning sensation and erythema, between the two groups (12 vs. 4 events)	(31)
Capsaicin	A pretest-post-test quasi-experimental study	9 out of 21 patients completed a 4-weeks trial. One area was treated with 0.025% capsaicin cream 4 times daily	All patients experienced an improvement in pruritus from moderate or severe at baseline to minimal or no pruritus even after 1-week application.	One case reported mild product-related burning sensation	(32)
Capsaicin	Randomized double-blinded crossover clinical trial	Either 0.03% capsaicin cream (17 cases) or placebo (17 cases) was applied to pruritic areas 4 times daily for 4 weeks. After stopped for 2 weeks, the two groups switched treatments and continued for another 4 weeks.	Pruritus scores were reduced from baseline 15.9 ± 6.3 to 2.5 ± 2.5 in capsaicin group at week 4. Pruritus scores were reduced from baseline 15.0 ± 6.0 to 7.2 ± 5.5 in placebo group at week 4	All cases experienced a burning sensation in the first week, followed by a decrease in the following week.	(33)
Gabapentin	Randomized, double-blind, vehicle-controlled study	15 patients were treated with 6% gabapentin cream once daily for 2 weeks, and controls were 15 patients treated with vehicle alone.	Gabapentin induced more significant reduction in VAS (4.6 vs. 2.6, *p* < 0.01)	No adverse events	(34)
Physiological lipids with endocannabinoids	A pretest-post-test quasi-experimental study	21 subjects were treated with a cream containing physiological lipids and endocannabinoids twice daily for 3 weeks.	VAS was decreased from 6.24 ± 2.19 to 1.29 ± 1.41; Dryness was decreased from 1.76 ± 0.94 to 0.19 ± 0.4	All subjects tolerated the product without any observed side effects.	(35)
Oat lotion	Randomized pretest-post-test quasi-experimental study	Oat skin protectant lotion was applied to the lower leg twice daily for 2 weeks. Untreated group served as controls. (40 patients per group)	Changes in median itching sensation were −2.5 in the treated group (*p* = 0.000), and 1 in the controls (*p* = 0.001)	NR	(36)
*Avena sativa*	Crossover randomized pretest-post-test quasi-experimental study	*Avena sativa* lotion was applied to the pruritic areas of 23 patients twice daily for 2 weeks.	VAS was decreased from 5.21 ± 1.69 to 4.10 ± 2.34, *p* < 0.01	NR	(37)
Vinegar	Crossover randomized pretest-post-test quasi-experimental study	5% vinegar was applied to the pruritic areas of 23 patients twice daily for 2 weeks.	VAS was decreased from 5.19 ± 1.88 to 3.73 ± 2.41, *P* < 0.001	NR	(37)
Urea plus dexpanthenol	A pretest-post-test quasi-experimental study	10% urea ISDIN^®^ plus dexpanthenol was applied to the arms and the legs of 15 patients with UP, twice daily for 28 days.	82% reduction in SRRC (*p* = 0.0001) and 94% reduction in itching score (*p* = 0.008)	Mild burning sensation in 1 case.	(38)
Pramoxine	Randomized, double-blind, controlled comparative trial	1% pramoxine lotion was applied twice daily to affected areas (13 cases) for 4 weeks. Cetaphil lotion was used as control product (14 cases).	Reductions in VAS in the control and pramoxine groups were 12% and 61%, respectively.	No adverse events	(39)
Sericin	Randomized, double-blind, placebo-controlled trial	8% sericin cream and cream base (placebo) on either the right or left extremities of 47 patients twice daily for 6 weeks.	Sericin increased the stratum corneum hydration on the arm and leg by 17% and 16%, respectively (*p* < 0.05 for both). VAS was decreased by 69% (*p* = 0.002). Placebo did not induce significant change.	NR	(40)
Cromolyn sodium	Randomized, double-blind, placebo-controlled trial	Either 4% cromolyn sodium or placebo was applied to the affected areas (30 patients per group) twice daily for 4 weeks	Placebo decreased VAS by 51%, and cromolyn by 88% (*p* < 0.05).	Six patients experienced a burning sensation at the beginning of the study, which gradually disappeared by the end of the trial.	(41)
Gamma-linolenic acid	Randomized, double-blind, placebo-controlled trial	Either GLA 2.2% cream or placebo-based cream applied to the entire body of 14 cases after taking a bath once daily and to pruritic sites thrice daily for 2 weeks.	Placebo decreased VAS from 80 to 75; GLA decreased VAS from 75 to 30 (*p* < 0.0001).	One subject had skin allergic reaction.	(42)
Mixture of peppermint oil and sunflower oil	A pretest-post-test quasi-experimental study	Mixture of peppermint oil and sunflower oil was applied to affected areas twice daily for 2 weeks (*n* = 30)	80% decrease in 5-D IS (*p* < 0.0001 vs. baseline)	Seven subjects were allergic to product.	(43)
Mixture of lavender and tea tree oil	A pretest-post-test quasi-experimental study	The arms of 13 patients were massaged 7 min with the 5% of the mixture in jojob oil and sweet almond oil three times per week for 4 weeks. 16 controls were without oil treatment	Oil treatment decreased pruritus intensity by 53%, while increasing skin hydration by 10% (*p* = 0.001 for both), with no changes in either pruritus or skin hydration in the controls	NR	(44)
A mixture of multiple ingredients	A pretest-post-test quasi-experimental study	Medi- Secure Atoderm Xereane was applied to the skin of 29 cases once or twice daily for 28 days.	Both pruritus severity and dermatological life quality index were markedly improved.	93% of cases rated the safety of the product as good to very good.	(45)
Water	Non-randomized pretest-post-test quasi-experimental study	10 patients with mild UP were treated with aqueous gel (80% of water) twice daily for 2 weeks. 10 untreated patients served as controls.	83% reduction in VAS and 90% improvement in skin dryness after 2-weeks treatment; 66% reduction in VAS and 55% improvement in skin dryness 2 weeks after stopping the treatment.	NR	(46)
Dead sea water	Randomized, double-blind, placebo-controlled trial	21 patients were treated with dead sea water lotion twice daily for 2 weeks. 20 patients treated without dead sea water (P1) and 24 patients treated without any active ingredients (P2) served as controls	Only P1 significantly improved itching score from the baseline. But the differences were not significant among three groups.	Two adverse events were reported in cases treated with dead sea water lotion	(47)
*Sambucus ebulus* fruit extract	Randomized, single-blind, placebo-controlled trial	31 patients were treated 2% of *Sambucus ebulus* fruit extract gel twice daily for 8 weeks, and 30 patients treated with placebo served as controls.	Significant reduction in itching scores, but not skin dryness, in both groups. No significant differences between the two groups.	One case was allergic to the test product	(48)
Urea	Randomized, double-blind, placebo-controlled trial	20% urea in NaPCA and vegetable oil was applied to 32 patients twice daily for 4 weeks. 33 patients treated with placebo served as controls	20% urea product significantly reduced VAS and increased stratum corneum hydration levels at both week 2 and 4 (*p* = 0.000 vs. placebo at both time points).	No adverse event.	(49)
Tacrolimus	A pretest-post-test quasi-experimental study	21 patients were treated with 0.1% tacrolimus twice daily for 3 weeks, followed by 0.03% tacrolimus for another 3 weeks.	Pruritus score decreased by 81%	Four cases experienced transient tingling and burning sensation in the 1st week, and one had skin rash after 1-week treatment. One had tingling over the entire trial period.	(50)
Tacrolimus	Short-term efficacy of tacrolimus ointment in severe uremic pruritus.	0.03% tacrolimus was applied twice daily for 7 days to the affected areas of 3 patients with severe UP.	Dramatical decrease in VAS	No adverse events	(51)
Tacrolimus	Randomized, double-blind, placebo-controlled trial	A randomized, vehicle-controlled study was carried out in 8 vehicle controls and 12 treated patients. One of the investigators applied 0.1% of tacrolimus to affected areas 3 times weekly and other applications were done by the patients at home twice daily for 4 weeks.	Reductions in VAS in vehicle and tacrolimus by 79% and 77%, respectively.	One case reported a significant burning sensation that subsided with continuous applications	(52)
Pimecrolimus	Randomized, double-blind, placebo-controlled trial	1% pimecrolimus was applied topically twice daily for 8 weeks. Placebo-treated served as controls. 30 patients per group	Both treatments significantly lowered VAS. No differences were observed between the two groups.	23 patients reported a burning sensation that gradually disappeared before the end of the trial	(53)

VRS, verbal rating scale; ISS, Itch Severity Scale; 5-D IS, 5-D itch scale; SRRC, scaling, roughness, redness and cracks; G/P, glycerol and paraffin; GLA, gamma-linolenic acid; PSQI-P, Pittsburgh Sleep Quality Index; Itchy QoL, Itchy Quality of Life; NR, no report.

## 2 Edible natural oils

Several edible oils have shown beneficial effects on uremic pruritus (UP). A randomized clinical study indicated that the topical application of sweet almond oil to itchy areas once daily for 1 week significantly improved pruritus severity and the Itchy Quality of Life (QoL) in patients undergoing hemodialysis (*p* < 0.01), with further improvement observed after a 2-week treatment (*p* < 0.001) (23). Similarly, topical applications of either virgin coconut oil or olive oil twice daily for 1 week markedly reduced the intensity of pruritus in patients with UP (25). Dill oil is another edible natural oil exhibiting anti-pruritic properties. Likewise, another randomized double-blind study demonstrated that either dill oil or sesame oil was applied topically to the itchy areas of UP patients twice daily for 1 month, followed by assessments of skin dryness, the Pittsburgh Sleep Quality Index, Duo’s Uremic Pruritus Severity Scale, and the Itchy QoL. The results showed that both oils improved the Itchy QoL and skin dryness compared to untreated controls. While dill oil significantly improved sleep quality scores, sesame oil did not show a similar effect when compared to untreated controls. A more profound improvement in pruritus severity was observed in the dill oil-treated group, suggesting that dill oil is more potent than sesame oil in alleviating pruritus (26).

In addition to pure oils, topical applications of diluted oils also relieve pruritus. For example, topical applications of 5% peppermint oil twice daily for 2 weeks reduced the intensity of pruritus by 58% and the duration of pruritus (hours/day) by 50%, alongside a 53% reduction in the 5-D itch scale. Interestingly, 5% peppermint oil is also effective for hepatic pruritus, with efficacy similar to that observed for UP (29). Notably, peppermint oil and sunflower oil may provide synergistic benefits for UP. A study involving 30 patients demonstrated that massaging the skin for 10 min with a mixture of these two oils twice daily for 2 weeks reduced pruritus intensity by 80% (43). Taken together, these findings indicate that certain edible natural oils can alleviate UP.

Regarding the underlying mechanisms through which these oils ameliorate UP, it is likely due to an increase in stratum corneum hydration, which levels are low in patients with end-stage renal (57). Stratum corneum hydration levels are negatively correlated with pruritus severity (58–60). Reduced stratum corneum hydration can lead to increased histamine and proinflammatory cytokines in the skin (61, 62), with both histamine and proinflammatory cytokines acting as mediators of pruritus (63). Some edible natural oils, such as violet oil, almond oil, and virgin coconut oil, can increase stratum corneum hydration levels (24, 64, 65). Additionally, certain oils, such as sunflower oil, olive oil, and coconut oil, exhibit anti-inflammatory properties (66). Thus, the inhibition of inflammation contributes to the beneficial effects of edible natural oils on UP.

## 3 Byproducts of crude oil

Both mineral oil and petrolatum, byproducts derived from crude oil refining, are widely used in skin care products. Clinical observations have demonstrated that topical mineral oil, petrolatum, or their combination with other ingredients can relieve UP. Lin et al. reported that topical applications of either chilled (10 °C–15 °C) or room-temperature mineral oil, applied at least once daily for 3 weeks, lowered pruritus severity by 38% and improved sleep quality by over 40% in a randomized clinical trial (17). Another randomized study showed that topical mineral oil applied three times daily for 4 weeks reduced pruritus severity by 43% (18). Similarly, topical chilled mineral oil not only improves pruritus and sleep but also enhances overall quality of life (19). Additionally, several randomized studies demonstrated the benefit of the combination of mineral oil and glycerol for UP (20–22). Surprisingly, petrolatum, a common humectant, did not improve the total 5-D itch scale score, although pruritus intensity was marginally improved following a 2-weeks treatment (10% reduction, *p* < 0.05 vs. pretreatment) (29). The benefits of mineral oil and petrolatum for UP are likely attributable to improved stratum corneum hydration. Since the permeability barrier is comparable between patients with UP and those without (55, 56), the improvement of epidermal permeability barrier function by petrolatum is unlikely to contribute to the alleviation of pruritus.

## 4 TRPV1 agonist

Transient receptor potential vanilloid 1 is a nonselective cation channel, which activation by noxious stimuli provokes sensation of pruritus. Capsaicin, a desensitizing TRPV1 agonist, inhibits pruritus induced by cytokines and histamine (67, 68). In humans with UP, pretreatment of the skin with 0.05% capsaicin reduced pruritus and wheal size in response to histamine. In contrast, 0.05% capsaicin did not affect serotonin-induced itching in either healthy subjects or patients with UP (69, 70). A randomized study involving 17 patients with UP showed that four times daily treatment with 0.025% capsaicin completely resolved pruritus in 5 patients and markedly relieved it in 9 patients. The relief of pruritus persisted for 4 weeks after the discontinuation of treatment (31). A 1-week treatment with 0.03% capsaicin significantly reduced the pruritus score (60% vs. 22% in vehicle-treated, *p* < 0.001). After 4 weeks of treatment in a randomized study, both capsaicin and vehicle lowered the pruritus score by 84% and 52%, respectively (*p* < 0.001) (33). Topical capsaicin also inhibits histamine-induced pruritus (71), but not serotonin-induced pruritus (69). Several randomized and non-randomized studies have also demonstrated that topical capsaicin is effective for UP (31–33). However, safety concern about capsaicin should be raised since it can decrease cell viability and increase IL-6 expression (72), in turn, potentially compromising epidermal function and negatively impacting pruritus. Capsaicin can also increase pain intensity and cause burning sensation (32, 73).

## 5 Others

### 5.1 Combination of the stratum corneum lipids and endocannabinoids

The mixture of stratum corneum lipids is known to improve epidermal permeability barrier function, enhance stratum corneum hydration, and inhibit inflammation (74–77), resulting in alleviation of pruritus. Similarly, endocannabinoids bind to their receptors (CB1R and CB2R) on keratinocytes, inhibiting inflammation, while the activation of these receptors (particularly CB1R) on peripheral nerves decreases pruritus, especially histaminergic pruritus (78–81). Therefore, the combination of stratum corneum lipids and endocannabinoids may serve as an optimal approach for managing pruritus. Indeed, a preliminary study demonstrated that 38% of patients with UP experienced complete relief from pruritus following the topical application of a cream containing both physiological lipids and endocannabinoids, applied twice daily for 3 weeks. Overall, pruritus severity (visual analog scale, VAS) decreased by 79%, while skin dryness improved by 89%. This effect persisted for 2 weeks after the treatment was discontinued (35). However, whether these benefits are attributed to the synergistic effect of these two components remains to be explored.

### 5.2 Combination of urea and dexpanthenol

Urea has been used in skincare products for years due to its ability to enhance epidermal permeability barrier function, increase stratum corneum hydration, and exert antimicrobial properties through stimulation of keratinocyte differentiation, epidermal lipid synthesis, antimicrobial peptide production, and aquaporin expression (82–84). Dexpanthenol, also known as vitamin B5, can be absorbed by the skin, where it is enzymatically converted to pantothenic acid, a constituent of coenzyme A, which catalyzes the synthesis of fatty acids and sphingolipids, thereby benefiting the epidermal permeability barrier and stratum corneum hydration (85, 86). Dexpanthenol-containing products have been used in the treatment of various inflammatory dermatoses, such as dermatitis and psoriasis (87, 88). Topical dexpanthenol, or panthenol (a derivative of pantothenic acid), inhibits cutaneous inflammation, alleviates pruritus, and enhances epidermal permeability barrier function and hydration (85, 86, 89–91). These effects suggest a potential benefit of urea and dexpanthenol for UP. A clinical trial involving 15 patients with UP demonstrated that scaling, roughness, redness, and cracks scale (SRRC) was decreased by 82%, while the itching score decreased by 94% after 4 weeks of twice-daily applications of 10% urea combined with dexpanthenol (38). Another study of 29 patients with UP showed that a formulation containing panthenol and other ingredients decreased SRRC and VAS by 83% and 49%, respectively, along with a 50% improvement in the Dermatological Life Quality Index (DLQI) after 4 weeks of treatment (70). A study involving 20 patients indicated that a 4-weeks treatment with 20% urea significantly increased stratum corneum hydration levels and reduced pruritus severity (49). Although the sample sizes were small, these results suggest that urea alone or in combination with dexpanthenol is effective for UP.

### 5.3 Heparinoid

Heparinoid-containing product (Hirudoid^®^, 0.3% of heparinoid) exhibits anti-inflammatory and moisturizing effects, as well as improves epidermal permeability barrier homeostasis in the skin (92–94). A multicenter, open-label, randomized study of 71 patients with UP showed that a 2-weeks treatment with Hirudoid^®^ significantly increased stratum corneum water content (*p* < 0.0001 vs. baseline). In contrast, pruritus severity (VAS) was decreased by 50%–60%, accompanied by a significant improvement in DQLI (95). These effects may be attributed to the inhibition of inflammation, as well as improvements in epidermal permeability barrier function and stratum corneum hydration.

### 5.4 Calcipotriol

Calcipotriol is a synthetic derivative of vitamin D. It has been shown to be effective in the management of inflammatory disorders, such as acne and psoriasis (96–99), partly through the stimulation of keratinocyte differentiation, inhibition of inflammation, and reduction of oxidative stress (100–103). A low dose of calcipotriol (0.05 microg/g) relieves pruritus in patients with dystrophic epidermolysis bullosa (104). Correspondingly, topical calcipotriol at 0.005% applied twice daily for 4 weeks reduced the VAS from 6.61 ± 2.49 to 1.45 ± 1.58, while increasing stratum corneum hydration levels from 14.56 ± 5.88 to 43.55 ± 7.91 in patients with UP (30). This randomized study clearly demonstrated the benefits of calcipotriol for both stratum corneum hydration and pruritus. However, calcipotriol can induce cutaneous inflammation (105, 106), which may exacerbate pruritus. Caution should be exercised when using calcipotriol for the treatment of UP.

### 5.5 Protein

Sericin, a water-soluble protein derived from the silkworm, exhibits multiple biological functions, including anti-inflammatory, antioxidant, and wound healing properties (107–110). Sericin also increases stratum corneum hydration levels, likely due to its high content of serine (33%), a natural moisture factor in the skin (111). Aramwit et al. treated one side of the extremities with 8% sericin and the contralateral side with a placebo twice daily for 6 weeks. Treatment with 8% sericin, but not the placebo, significantly increased stratum corneum hydration levels on both the arm and leg (*p* < 0.05 for both). In parallel, the Visual Analog Scale (VAS) decreased by 69% (*p* = 0.002) (40), suggesting that sericin may be a valuable treatment regimen for uremic pruritus.

### 5.6 Local anesthetics

Pramoxine is a topical local anesthetic that has been shown to have antipruritic properties through inhibition of voltage-gated sodium channels, consequently leading to blockade of signal transmission (112). A group of women with dry, itchy skin reported significant improvements in both pruritus severity and skin dryness following the topical application of a combination of 12% lactic acid and 1% pramoxine hydrochloride twice daily for 1 day, with further improvements observed after 7 days of treatment (113). In contrast to this open label, investigator-blinded study, a randomized study showed that twice-daily treatment with 1% pramoxine for 4 weeks lowered pruritus severity by 49%, without significantly improving stratum corneum hydration levels compared with the controls (39). Thus, the improvement in stratum corneum hydration observed in some pramoxine-containing products may be attributable to other ingredients rather than pramoxine itself in the formulation.

### 5.7 Natural extracts and their analogs

Oat (*Avena sativa*) exhibits antioxidant and anti-inflammatory property (114). It also contains high content of essential unsaturated fatty acid (115), which activate peroxisome proliferator activated receptors, resulting in stimulation of keratinocyte differentiation and lipid production, consequently improving epidermal permeability barrier function (116, 117). These benefits suggest that oats have antipruritic potential. Accordingly, two separate randomized studies demonstrated that topical application of oat lotion twice daily for 2 weeks significantly reduced pruritus severity by 20%–27% in patients with UP (36, 37). Cromolyn sodium is a synthetic analog of khellin, a natural compound from *Ammi visnaga*, and functions as mast cell stabilizer preventing the release of inflammatory mediators, including histamine (118). A placebo-controlled study demonstrated that applying 4% cromolyn sodium twice daily for 4 weeks reduced pruritus severity (VAS) by 88% (*p* < 0.05), compared to 52% for placebo (41). In addition, 7-min massaging with 5% of a mixture (lavender and tea tree oil) in jojob oil and sweet almond oil three times per week for 4 weeks decreased pruritus score by 53% (*p* = 0.001 vs. both baseline and untreated controls), while increasing stratum corneum hydration levels by 10% (*p* < 0.01 vs. baseline; *p* = 0.001 vs. untreated controls) (44). However, it is unclear whether these beneficial effects are attributable to the mixture of lavender and tea tree oil, the vehicle (jojoba oil and sweet almond oil), or both. A formulation consisting of 80% water and 20% other ingredients (including aloe vera extract, silk powder, naturally derived vitamin E, and squalane) reduced pruritus severity by 83% and skin dryness by 90% after a 2-weeks, twice-daily treatment (46). Again, it remains unknown which ingredient(s) contributes to these benefits. Interestingly, topical applications of vinegar (0.3%) twice daily for 2 weeks reduced pruritus severity score by 28% (*p* < 0.001 vs. baseline) (37), suggesting a pathogenic role elevated skin surface pH in the development of UP. Previous studies reveled that skin surface pH is higher in hemodialysis patients than in the controls (60). The increased pH can activate protease activated receptor 2 (PAR2), resulting pruritus (119–123). Conversely, acidification of the stratum corneum inhibits cutaneous inflammation and PAR2 expression, at least, in mice (124). Inhibition of PAR2 relieves pruritus (121). Thus, acidification of stratum corneum may serve as an alternative approach for the management of UP.

Other topical emollients have also been shown to be effective for UP. For example, gabapentin, an anticonvulsant, bind to a specific site (α2-δ) on voltage-gated calcium channels and possibly other proteins, decreasing nerve excitability (125). It is used to treat seizures and nerve pain. A vehicle-controlled study showed that topical 6% gabapentin once daily for 2 weeks significantly reduced pruritus severity (VAS) (78% vs. 42% for vehicle, *p* < 0.01) (34). The outcomes of calcineurin inhibitors for UP are inclusive. Two studies comparing pre- and post-treatment found that tacrolimus cream ameliorated pruritus severity (50, 51). However, two other placebo-controlled studies did not show significant differences in pruritus improvement between the test product and placebo (52, 53). Together, this line of evidence does not support the beneficial effects of calcineurin inhibitors for UP. These negative results of calcineurin inhibitors can be attributed to their adverse effects on epidermal function. While these inhibitors ameliorate inflammation, study has also shown that topical tacrolimus compromises epidermal permeability barrier function. Defective permeability barrier can induce and/or exacerbate cutaneous inflammation, increase epidermal nerve density and skin sensitivity to stimuli (126, 127), resulting in pruritus. Hence, calcineurin inhibitors may not be suitable for treating pruritus in some cases, especially with long-term use.

In Summary, pruritus is common in patients with end-stage renal dysfunction. Treatment of uremic pruritus (UP) remains a significant challenge, owing in large part to uncertainty regarding its pathogenesis. Current management strategies include topical therapies, systemic therapies, phototherapy, and nonpharmacologic modalities such as acupressure. Among topical treatments, most evidence supports the benefits of emollients with moisturizing effects, which align with the role of low stratum corneum hydration levels in pruritus. Certain emollients containing mixtures of stratum corneum lipids have shown efficacy and favorable safety for long-term use, extrapolated from their established utility in other pruritic dermatoses such as atopic dermatitis, age-related xerosis, and psoriasis. Given that cutaneous proteases function optimally near neutral pH (128), and that xerosis contributes to both pruritus and inflammation, acidic moisturizers represent a mechanistically plausible intervention for UP. Additionally, emollients with both moisturizing and anti-inflammatory properties may be more effective than moisturizers alone. These hypotheses, however, require validation in adequately powered, well-designed clinical trials.

This review has several limitations. A large portion of the studies was not randomized and conducted in small group of patients. More randomized, placebo-controlled studies in larger cohort are needed to validate the efficacy of emollients in treating UP. Moreover, the literature search was conducted exclusively on PubMed and Google Scholar. Studies from other databases and those published in languages other than English were not included in this review. Consequently, some evidence may not be covered in this review. A thorough literature search, including sources from other databases and in other languages can provide additional valuable evidence regarding the role of emollients in uremic pruritus. Since the pathogenesis of UP may vary among individuals, emollients may not be effective for everyone with UP. For example, if dry skin is not the key contributor in some cases, improving stratum corneum alone may not alleviate UP. Thus, an individualized approach should be considered in the management of UP.
